# Supplementary material for: Psychometric properties of the Patient Reported Outcomes, Burdens and Experiences (PROBE) questionnaire
Source: BMJ Open. 2018 Aug 8;8(8):e021900. doi: 10.1136/bmjopen-2018-021900 (PMC6089314; doi:10.1136/bmjopen-2018-021900)
Supplement: Author's manuscript [file bmjopen-2018-021900.draft_revisions.pdf]

BMJ Open

BMJ Open is committed to open peer review. As part of this commitment we make the peer review history of every article we publish publicly available.

When an article is published we post the peer reviewers' comments and the authors' responses online. We also post the versions of the paper that were used during peer review. These are the versions that the peer review comments apply to.

The versions of the paper that follow are the versions that were submitted during the peer review process. They are not the versions of record or the final published versions. They should not be cited or distributed as the published version of this manuscript.

BMJ Open is an open access journal and the full, final, typeset and author-corrected version of record of the manuscript is available on our site with no access controls, subscription charges or pay-per-view fees (http://bmjopen.bmj.com).

If you have any questions on BMJ Open's open peer review process please email info.bmjopen@bmj.com

BMJ Open

Psychometric properties of the Patient Reported Outcomes Burdens and Experiences (PROBE) Questionnaire

Journal:	*BMJ Open*
Manuscript ID	bmjopen-2018-021900
Article Type:	Research
Date Submitted by the Author:	26-Jan-2018
Complete List of Authors:	Chai-Adisaksopha, Chatree; McMaster University, Medicine; McMaster University, Health Research Methods, Evidence, and Impact<br>Skinner, Mark; Institute for Policy Advancement Ltd<br>Curtis, Randall; Factor VIII Computing<br>Frick, Neil; National Hemophilia Foundation<br>Nichol, Michael; University of Southern California Sol Price School of Public Policy<br>Noone, Declan; Irish Haemophilia Society<br>O'Mahony, Brian ; Irish Haemophilia Society; Trinity College Dublin<br>Page, David; Canadian Hemophilia Society<br>Stonebraker, Jeffrey ; North Carolina State University Poole College of Management<br>Thabane, Lehana; McMaster University, Health Research Methods, Evidence, and Impact; St Joseph's Healthcare, Biostatistics Unit<br>Crowther, Mark; McMaster University, Canada<br>Iorio, Alfonso; McMaster University, Clinical Epidemiology and Biostatistics
Keywords:	validity, hemophilia, patient reported outcome, quality of life, questionnaire

SCHOLARONE™
Manuscripts

Psychometric properties of the Patient Reported Outcomes Burdens and Experiences (PROBE) Questionnaire

Authors

Chatree Chai-Adisaksopha[1,2], Mark Skinner[3], Randall Curtis[4], Neil Frick5, Michael B. Nichol[6], Declan Noone[7], Brian O'Mahony[7,8], David Page[9], Jeffrey Stonebraker[10], Lehana Thabane[2,11], Mark Crowther[1,2] and Alfonso Iorio[1,2]

Authors' affiliations

[1]Department of Medicine, McMaster University, Hamilton, ON, Canada

[2]Department of Health Research Methods, Evidence, and Impact, McMaster University, Hamilton, Canada

[3]Institute for Policy Advancement Ltd, Washington, DC, US

[4]Factor VIII Computing, Berkeley, US

[5]National Hemophilia Foundation, New York, US

[6]Sol Price School of Public Policy, University of Southern California, Los Angeles, US

[7]Irish Haemophilia Society, Dublin, Ireland

[8]Trinity College Dublin, Dublin, Ireland

[9]Canadian Hemophilia Society, Montreal, Canada

[10]Poole College of Management, North Carolina State University, Raleigh, US

[11]Biostatistics Unit, St Joseph's Healthcare, Hamilton, Canada

For peer review only

Sources of financial support: PROBE is an independent investigator led research project with grant / research support from: Baxalta, now part of Shire; Bayer; Bioverativ; CSL Behring; Novo Nordisk, Roche and Sobi with administrative support provided by the US National Hemophilia Foundation.

Acknowledgements: The PROBE investigators wish to thank the hemophilia patient organizations that have participated in the project and two anonymous reviewers for significant contribution to the quality and clarity of the final version of this manuscript, including some key methodological references.

Running title: Psychometric properties of PROBE

Keywords: validity, hemophilia, patient reported outcome, quality of life, questionnaire

Word count: Abstract 245, Text 3715

Table and Figure: 6 tables, 2 figures

Correspondence:

Mark W. Skinner

1155 23rd Street NW #3A

Washington DC 20037 USA

Email: mskinnerdc@gmail.com

Tel:. +1-202-253-8342

Fax: +1-202-296-1352

For peer review only

Abstract

Objective: To assess the psychometric properties of The Patient Reported Outcomes, Burdens and Experiences (PROBE) questionnaire.

Methods: This study was a cross-sectional, multi-national study. Participants were enrolled if they were 10 years or older and patients with hemophilia A or B or people without bleeding disorder. Participants were invited through non-governmental patient organizations in 21 countries between 04/08/2015 and 12/28/2015.The following psychometric properties: missing data, floor and ceiling effects, exploratory factor analysis, and internal consistency reliability were examined. A PROBE Score was derived and assessed for its convergent and known groups validity.

Results: The study analyzed the data on 916 participants with median age of 37.0 (interquartile range 27.0 to 48.0) years, 74.8% male. In the domain assessing patient reported outcomes, more than 15% of participants presented a ceiling effect for all items but two, and a floor effect for one item. Factor analysis identified two factors explaining the majority of the variance. Cronbach's alpha coefficient indicated good internal consistency reliability (0.84). PROBE items showed moderate to strong correlations with corresponding EQ-5D-5L domains. The PROBE Score has a strong correlation (r=0.67) with EQ-5D-5L utility index score. The PROBE Score has a known groups validity among various groups.

Conclusions: The results of this study suggest that PROBE is a valid questionnaire for evaluating PROs in people with hemophilia, as well as control population. The known-group property of PROBE will allow its use in future clinical trials, longitudinal studies, health technology assessment studies, routine clinical care or registries.

For peer review only

Trial registration: NCT02439710

For peer review only

Strengths and limitation

- The PROBE questionnaire includes items assessing comprehensive outcomes that are relevant to patients.
- The psychometric analyses demonstrate the validity and internal consistency of the Patient Reported Outcomes Burdens and Experiences (PROBE) Questionnaire.
- This study was conducted in a large sample of patients with hemophilia and participant without bleeding disorders from multiple countries.
- The responsiveness of the measurement was not investigated win this current study.

For peer review only

Background

Hemophilia is an inherited X-linked recessive bleeding disorder characterized by the reduction or absence of blood coagulation factor (F) VIII (hemophilia A) or FIX (hemophilia B). Severity of hemophilia is categorized by the baseline factor level (mild; factor level >0.05 to <0.40 IU/ml, moderate; factor level 0.01-0.05 IU/ml and severe; factor level<0.01 IU/ml) [1]. Coagulation deficiency renders patients prone to abnormal bleeding. Symptoms of hemophilia vary depending on the severity of hemophilia, mechanism and severity of injury and affected organs. People with hemophilia (PWH) commonly present with hemarthrosis, gastrointestinal or genitourinary tract bleeding, intramuscular bleeding or intracranial bleeding [2-6].

Life expectancy of PWH substantially improved with factor replacement therapy [7]. However, PWH who live longer encounter more chronic complications from both hemophilia-related conditions and degenerative diseases that occur in normal population. Chronic degenerative joint diseases are found in 90% of PWH by the second or third decade of life [8]. PWH with recurrent joint bleeding suffer from chronic pain, limitation of range of motion and disability [9]. Human immunodeficiency virus (HIV) and hepatitis C virus (HCV) infections are prevalent among PWH prior to the implementation of intensive viral screening in plasma-derived factor concentrates and the use of recombinant factor concentrates [10]. One of the major consequences of chronic HCV infection is cirrhosis, resulting in end-stage liver disease which is the most common cause of death in PWH [10]. Moreover, 43% of cancers diagnosed in PWH were related to HCV infection [11]. Aged PWH are also affected by cardiovascular diseases. A retrospective study using an administrative database of 3,422 males with hemophilia reported a prevalence of ischemic heart disease of 15% in PWH older than 60 years [12]. Risk factors of cardiovascular disease in PWH are equivalent to patients without hemophilia [13]. These long-

For peer review only

term complications of hemophilia directly impact on health-related quality of life (HRQoL) in PWH [14].

Patient reported outcomes (PROs) are defined as any reports of status of patients' health conditions that come directly from the patients without interpretation by clinicians or anyone else [15]. PROs provide data that obtained from patients including symptoms, frequency of symptoms, severity of symptoms, impact of disease on daily life, disability and perfection of patients toward diseases and treatments [16]. Thus, PROs have been increasingly valued by researchers, stakeholders, policy makers and health technology assessment agencies [17-20]. Recently, the International Society for Pharmacoeconomic and Outcomes Research (ISPOR) Clinical Outcome Assessment Emerging Good Practices Task Force published the Patient-reported outcome and observer-reported outcome assessment in rare disease clinical trials [21]. This report demonstrated the challenges of assessing patient-reported outcome in rare diseases, for instance, heterogeneity of disease severity and patient experience or understanding treatment benefit from patients' perspective. Hemophilia, which is a rare bleeding disorder, exhibits various disease severity. Moreover, patients' perspective on their symptom may be dissimilarly influenced by age, co-morbid disease, inhibitor status, current treatment or progression of symptoms. Therefore, a hemophilia-specific PRO measure is essential for assessing outcomes in this patient population.

The Patient Reported Outcomes, Burdens and Experiences (PROBE) Project is a patient-lead research initiative. The main objectives of the PROBE Project are to develop a standardised PRO questionnaire and to develop a dedicated research network to generate and continuously update PROBE reference data. The rationale, research group establishment and PRO questionnaire development [22] has been previously reported. The feasibility study of the

For peer review only

PROBE questionnaire was conducted in collaborations with non-governmental hemophilia patient organizations (NGOs) in 17 countries. Previously reported results demonstrated that the burden of the PROBE questionnaire implementation was minimal and the time required to complete the questionnaire was less than 15 minutes for over 75% of participants [22]. The objective of the current study is to assess the psychometric properties of the PROBE questionnaire.

For peer review only

Methods

Participant enrollment and study procedure

This study was designed as a cross-sectional assessment. Participants were enrolled through NGOs from 1/27/2016 to 2/23/2017. Participants were recruited if they were more than 10 years old and they were either PWH (hemophilia A or hemophilia B) or controls (participants without bleeding disorders). Participants were instructed to complete the questionnaire for themselves, and parents or caregivers not to answer for their child. Although collected as part of the study, participants who identified themselves as carriers of hemophilia were excluded from the analysis. Patients with other bleeding disorders or an unknown bleeding disorder were also excluded.

The participating NGOs distributed the PROBE questionnaires through mail, e-mail, in-person meetings or a combination of methods. The PROBE questionnaire was available in 18 languages with localized language versions in both paper- and web-based format.

Ethical approval

Patients' identifier or personal information were not collected as part of the study. Data were collected as anonymous individuals, and study data were transferred and stored at McMaster University. Ethical approval was obtained from the Hamilton Integrated Research Ethics Boards. Additional local review ethical board approval was obtained when requested by the local regulation.

PROBE questionnaire

The detail of questionnaire development and feasibility study was described elsewhere [22]. The PROBE questionnaire is organized in 4 sections, comprising 29 questions. Sections are numbered following the order of presentation in the questionnaire. PROBE PRO domains are

For peer review only

covered in Section II. The questions in Section I and III do not cover PRO domains. Only PWH are expected to complete Section III, whereas every participant completes Sections I, II and IV. Section I contains 7 questions pertaining to demographic data (country, gender, diagnosis of hemophilia or absence of a bleeding disorder, year of birth, body weight, age first started and finished school, marital status and children). Section II contains 9 questions pertaining to PROs, including general health issues, use of mobility aids or assistive devices, pain (including acute, chronic, and pain medications), daily activities, current work or student status, surgeries or procedures, and co-morbid diseases. Section III contains 12 questions pertaining to clinical aspects of hemophilia (severity of hemophilia, inhibitor status, bleeding history, hemophilia care, treatment regimen, target joints, joint bleeding, range of motion and life- or limb-threatening bleeds). Section IV contains the EuroQol five dimension 5-level instrument (EQ-5D-5L) [23], consisting of questions regarding mobility, self-care, usual activities, pain or discomfort and anxiety or depression, and the EuroQol visual analog scale (EQ-VAS) of global health [23] were incorporated in the PROBE questionnaire with permission.

Item scaling and PROBE score calculation

PROs were evaluated only in Section II. The calculation of the PROBE score was based on multiattribute value functions [24 25]. The assessed scores (X_i) were converted to returns-to-scale score (V_iX_i), given that $0 \leq V_i(X_i) \leq 1$. Q.8 which had a dichotomous response (0 = no, 1 = yes) produce dichotomous score of 0 and 1. Two questions (Q.10 and Q.15) asked for frequency of the use of pain medication(s) and number of surgeries or invasive procedures. The 6- and 7-level Likert scales from these two questions were converted to a returns-to-scale score, ranging from 0 to 1. The number of days absent from work or school (Q.14) was converted to returns-to-scale score by dividing by 366. Questions regarding mobility aids,

For peer review only - http://bmjopen.bmj.com/site/about/guidelines.xhtml

acute pain, chronic pain and co-morbid diseases (Q.9, Q.11, Q.12, Q.13 and Q.16) had multiple choices. The scales for these items were calculated based on the cumulative number of choices checked. We apply weight for subitems in each question (if needed). The final score was calculated by summing all of the 11 items scores from the 9 questions using additive value function and then scaled so the PROBE Score ranged from 0 to 1 (higher value indicates better health status).

Data analyses

Descriptive statistics

Demographic data of study participants were summarized using mean with corresponding standard deviation (SD) or median and quartile range as appropriate. Categorical data were summarized using numbers and percentages. Participants who did not respond in Q.3 (disease status; hemophilia A, hemophilia B, hemophilia carrier, other bleeding disorders or no bleeding disorder) were excluded from the analysis. An item distribution analysis to evaluate the proportion of missing data was performed. Floor and ceiling effects were evaluated by the proportion of respondents with scores at floor (minimum score) and ceiling (maximum score), respectively.

Psychometric analyses

Face and content validity were assessed and reported previously [22]. Test-retest reliability analyses of the PROBE questionnaire were reported elsewhere [26]. In the current study, the following psychometric analyses were carried out.

Factor analysis

An exploratory factor analysis of 9 questions, pertaining to the PROs (Section II). Principal component factor analysis was conducted with oblique rotation method was performed.

For peer review only

Investigators made *a priori* decision to retain all factors that had eigenvalues of 1.0 or greater, according to Kaiser criterion [27]. A scree plot was generated. The percentage of variance on the items that were explained by the factors was evaluated. Higher percentage indicated strong influence of the factors. The regression coefficients (factor loadings) of the item responses on the retaining factors after factor rotation was calculated.

Internal consistency reliability

An analysis to confirm the precision of the scale based on the intercorrelations of the items evaluating the same construct was conducted. We hypothesized that the questions asking about pain and the use of medications (Q.10-Q.13) were correlated. Cronbach's alpha was used to determine the correlation between items. Cronbach's alpha coefficient greater than 0.7 was considered to indicate acceptable reliability [28].

Convergent validity

The convergent validity of the items in the same construct with the existing, standardised questionnaire were assessed. Specifically, we hypothesized that the items asking about the use of mobility aids and assistive devices correlated with the mobility domain of EQ-5D-5L; the items asking about the use of pain medication, acute and chronic pain (Q.10, Q.11 and Q.12) correlated with pain and discomfort domain of EQ-5D-5L; the items asking about activities of daily living (Q.13) correlated with the self-care and usual activity domains of EQ-5D-5L. The correlation between EQ-5D-5L utility index score and the PROBE Score was assessed. Correlation coefficient (r) was interpreted as the followings , r 0.20-0.39; weak correlation; r 0.40-0.59, moderate correlation; r 0.60-0.79, strong correlation; and r 0.80-1.00, very strong correlation [29].

Known groups validity

For peer review only

The ability of the PROBE questionnaire to determine the differences between known subgroups was assessed. Participants were classified into groups, according to information collected in Section III, as diagnosis (hemophilia or non-hemophilia), severity of hemophilia (mild, moderate or severe), current inhibitor status (yes or no), number of bleeds in the past year (categorical variable), bleed in the past two weeks (yes, no), presence of target joint (yes, no), limitation of range of motion of the joints (yes, no) and life- or limb-threatening bleeding in the past year (yes, no). The PROBE Scores were compared between subgroups using t-test or one-way ANOVA for the univariate analysis, as appropriate. A priori hypotheses included PWH (as compared to participants without bleeding disorders), patients with severe hemophilia (as compared to mild and moderate hemophilia), patients with current inhibitor (as compared to those without an inhibitor), patients with greater numbers of bleeding, patients who had recent bleeding within the past 2 weeks (as compared to those without), patients with presence of target joint(s) (as compared to those without), patients who had reduced range of motion of any joints (as compared to those without) and patients who had life- or limb-threatening bleeding in the past year (as compared to those without) had worse PROBE scores. The multivariable analysis of the known group validity was conducted using a linear regression. The regression model included age and gender of participants in the analysis. Regression coefficients with corresponding 95% CI were reported. P-value less than 0.05 was considered statistically significant.

Results

Participants' demographic data

Since inception, NGOs from 21 countries have participated in the PROBE project. Figure 1 demonstrates the flow of participant selection who participated in this phase of research. There were 1287 participants who responded to the questionnaire. After excluding hemophilia carriers, other bleeding disorders and missing value, the analysis included 916 participants. Demographic data is shown in Table 1. Median age of PWHs was lower than that of controls, 33 (quartile 1, quartile 3 of 24, 46) vs 43 (quartile 1, quartile 3 of 34, 54) years. The proportion of male participants in hemophilia group was greater than those in control group (93.7% vs 6.4%). Among hemophilia patients, most had severe hemophilia. Seventeen participants (2.6%) of PWH had an inhibitor. during study period.

Descriptive analysis

Table 2 demonstrates item distribution and missing data. Ceiling effect greater than 15% was observed in all but one item (the use of pain medications) in Section II. Similarly, ceiling effect greater than 15% was observed in all domains of EQ-5D-5L. Floor effect greater than 15% was found in four items (problems related to health, bleeding in the past 12 months, limitation of range of motion and life- or limb-threatening bleeding). Missing data was 0% to 21.8% in Section II, 18.2% to 49.4% in Section III and 21.6% to 22.9% in Section IV. The median PROBE Score across all participants was 0.78 (mean=0.76, SD=0.16, minimum=0.26 and maximum=0.99).

Psychometric analyses

Exploratory factor analysis

For peer review only

The principal component factor analysis of the 9 questions (11 items) pertaining to the PROs was carried out. The scree plot demonstrated two factors with eigenvalue greater than 1.0 (Figure 2). These two factors were retained for the following analyses. Cumulatively, the combination of two factors explained 50.6% of the variance. Table 3 demonstrates factor loadings based on two factors. The items were grouped per factor with their maximum loading (bold).

Factor 1 appears to be the most influential, explaining 40.8% of the variance. There were 8 items contained in this factor (problems related to health, mobility aids or assistive devices, use of pain medications, activities and interference related to acute pain, activities and interference related to chronic pain, activities of daily living, and work/school life). Factor 2 explained 9.8% of the variance, and contained two items (joint surgery or procedure and comorbid disease). All items in the each factor had acceptable factor loadings ($r \geq 0.3$) [30].

Internal consistency reliability

The internal consistency reliability was carried out using Cronbach's alpha. An analysis on pain-related items was performed. The Cronbach's alpha coefficient was acceptable at 0.84.

Convergent validity

Table 4 shows the correlation coefficients between PROBE items and EQ-5D-5L. The results showed that Q. 3 (the use of mobility aids and assistive devices) had a moderate correlation with mobility domain of EQ-5D-5L (r=0.42). The pain and discomfort domain of EQ-5D-5L had a moderate to strong correlation with most of the pain related items of the PROBE questionnaire (r=0.55 for pain medication, 0.42 for acute pain occurrence, 0.39 for acute pain interference, 0.56 for chronic pain occurrence and 0.57 for chronic pain interference). Item related to activities of daily living had a strong correlation with the self care and usual

For peer review only - http://bmjopen.bmj.com/site/about/guidelines.xhtml

activities domain (r=0.65 and 0.71, respectively). The PROBE score had a strong correlation with the EQ-5D-5L utility index score (r=0.67).

Known groups validity

The regression coefficients of each *a priori* variable and the PROBE Score were demonstrated in Table 5. Participants without a bleeding disorder had a significantly higher PROBE Score when compared with PWH (mean score (SD), 0.87 (0.11) vs 0.71 (0.16), P<0.001). PWH with mild to moderate hemophilia had a slightly higher PROBE Score (mean 0.71, SD 0.16) than severe PWH (mean 0.70, SD 0.16), PWH who had a greater number of bleeding episodes had a significantly lower PROBE Score when compared to those who had less frequent bleeding (P<0.001). Patients who reported bleeding in the past two weeks had a significantly lower PROBE score (mean 0.67, SD 0.15) than those without (mean 0.76, SD 0.15). Patients who reported the presence of any target joints had a significantly lower PROBE score (mean 0.68, SD 0.15) when compared to those who did not (mean 0.78, SD 0.16). Patients who reported three or more spontaneous joint bleeds in the past 6 months had significantly lower PROBE score (mean 0.66, SD 0.14) than those who did not report (mean 0.73, SD 0.14). Patients with reduced range of motion of any joints had a significantly lower PROBE score (mean 0.68, SD 0.14) as compared to those without (mean 0.73, SD 0.15). Patients who previously had life- or limb-threatening bleeding in the past year had a significantly lower PROBE Score (mean 0.62, SD 0.16) when compared to those who did not (mean 0.72, SD 0.15). Table 6 demonstrates multivariable analysis. The findings from multivariable analysis did not change much after adjusting for age and sex.

For peer review only

Discussion

The psychometric properties of the PROBE questionnaire have been assessed, and found that the PROBE questionnaire has a strong internal consistency, robust convergent validity and excellent differentiation properties between known groups. We believe these characteristics, jointly with the availability of country specific reference ranges and low impact on NGO resources and time required by the patients make the PROBE questionnaire a tool with great potential for efficient PROs collection in clinical and comparative effectiveness research, and for advocacy purposes.

As demonstrated by factor analysis, the core of PROBE revolves around two factors, explaining the majority of the variance in responses. The most influential factor was pain, followed by use of mobility aids or assistive device (complemented by work or school absent days), and comorbidity. No surprise these three elements explain 50% of the variance among different participants: the novelty of PROBE is summarizing the assessment of these 3 domains in a lightweight set of questions for which excellent internal consistency was demonstrated.

The convergent validity analysis showed moderate to strong correlation between PROBE and EQ-5D-5L items, with lower correlations for items concerning pain (r ranged from 0.39 to 0.57). Whereas the overall convergence with EQ-5D-5L was confirmed, and was intentionally sought to ensure maximizing external validity and efficiency for cross-disease comparisons. The pain related questions in the PROBE questionnaire are related to different aspects (when the pain occurred..., if the pain interfered with any of following…) than EQ-5D-5L [31]. From this perspective, PROBE might be seen as a new hybrid PRO tool, sharing some properties of a generic and some of a disease specific tool. The total PROBE score has a strong correlation

For peer review only

with the utility index score of the EQ-5D-5L, both in patients (r=0.57), and controls (r=0.53), but explores a more specific set of subdomains.

The most important result of this analysis is the demonstration of the discriminative property of the PROBE questionnaire and score. In known group validity analysis, PWH had significantly lower PROBE Score when compared to the control population (participants without hemophilia). Patients with more frequent bleed, target joint, reduced range of motion and previous life- or limb-threatening bleed were demonstrated with a lower PROBE score (indicating worse health status).

The investigators did not observe a significant difference of the total PROBE scores among severity of disease, as well as, current inhibitor status. This outcome may be confounded by bleeding phenotype and joint status. It has been shown that the presence of inhibitor has negative impact on health-related quality of life in PWH [32]. The regression analysis in this present study revealed that numbers of bleeding, presence of target joint(s) and limitation of range of motion of any joints, not inhibitor status, were associated with worse health status. There have been studies that reported the negative health-related quality of life in hemophilia patients with inhibitor who had poor orthopedic joint score, who had acute bleeding and who had more frequent bleeding [33-35]. It is important to note that there are relatively a small number of patients with mild-moderate diseases (8.8% and 14.3%, respectively) and those with current inhibitors (4.1%) in this study. The association between inhibitor status and health status of PWH warrant further studies with adequate power.

The PROBE Project has several strengths. First, participants were recruited from21 countries involving 6 regions of the world. The finding of this study is therefore internationally generalizable regardless of languages and cultures. Second, both PWH and participants

For peer review only - http://bmjopen.bmj.com/site/about/guidelines.xhtml

without bleeding disorders were recruited, asked PRO questions meaningful to both, and derived a PROBE score applicable to both. Therefore, we were able to compare the health status across health-specific conditions (hemophilia vs non-hemophilia in this study). There is a potential role for the use of the PROBE questionnaire to compare health status between PWH with any other diseases that share common features, e.g. von Willebrand disease, rheumatoid arthritis or osteoarthritis. Third, both school-aged and adult participants were included. The work or school life was assessed in the same manner. As a result, the PROBE questionnaire is valid to implement in participants in all age groups (starting at the not-yet defined age when one is able to comprehend the questionnaire). Third, the questions in the PROBE questionnaire included a standardized observation period in each question stem, generally the past 12 months. This is helpful for participants to respond to each item closest to their actual health condition in a specific time frame.

This PROBE Project also has some limitations, the first being that responsiveness of the PROBE Score has not been validated currently. This study was conducted with a cross-sectional study design. This means participants responded to the questionnaire at a single time. Assessing responsiveness requires a more complicated and demanding study design, which will be addressed in the future. Second, the observation period in the items was up to 12 months. Whereas this was chosen to maximize capturing the impact of rare events, it might introduce recall bias in some participants. Third, a ceiling effect was observed for all except one item concerning PRO, as well as, all EQ-5D-5L items. The recent study regarding floor and ceiling effects of the EQ-5D-5L in 996 English general population showed that 47.6% of respondents reported the best possible heath state (ceiling effect) [36]. In addition, the ceiling effects ranged from 58.4% to 90.8% in the subdomains [36]. The floor effects in the study were

For peer review only

relatively lower than the previous reports [36], probably because sicker participants (PWH) were included

For peer review only

Conclusions

The properties of the PROBE questionnaire are suited for differentiating PWH with better or worse health status. The immediate use of the PROBE score based on these results would be in cross-sectional comparisons among different settings, e.g. those defined by different levels of access to care. Future applications, as assessing treatment effect in clinical trials, or monitoring patients' health status over time in longitudinal observational studies will enable us to define the responsiveness properties of PROBE to meaningful treatment and disease changes over time.

For peer review only

List of abbreviations

PROBE: Psychometric properties of the Patient Reported Outcomes Burdens and Experiences; EQ-5D-5L: EuroQol five dimension 5-level instrument; F:factor; EQ-VAS: EuroQol visual analog scale; PWH: people with hemophilia; HIV: Human immunodeficiency virus; HCV: hepatitis C virus; HRQoL: health-related quality of life; PRO: Patient reported outcome; ISPOR: International Society for Pharmacoeconomic and Outcomes Research; NGO: non-governmental organization; SD: standard deviation; ANOVA; analysis of variance

Declarations

Ethics approval and consent to participate

Ethical approval was obtained from the Hamilton Integrated Research Ethics Boards. Additional local review ethical board approval was obtained when requested by the local regulation.

Consent for publication

Not Applicable.

Availability of data and material

Not Applicable.

Competing interests

CC, LT, MAC have no potential conflict of interest. Investigators received grants from Baxalta, now part of Shire; Bayer; Bioverativ; CSL Behring, Novo Nordisk; Roche; and Sobi and non-financial support from the US National Hemophilia Foundation.

For peer review only

Funding

PROBE is an independent investigator led research project with grant / research support from: Baxalta, now part of Shire; Bayer; Bioverativ; CSL Behring; Novo Nordisk, Roche and Sobi with administrative support provided by the US National Hemophilia Foundation.

Acknowledgements: The PROBE investigators wish to thank the hemophilia patient organizations that have participated in the project and two anonymous reviewers for significant contribution to the quality and clarity of the final version of this manuscript, including some key methodological references.

Authors' contributions

MS, AI, RC, NF, MN, DN, BOM, DP and JS conceptualized the study. CC and LT performed data collection and statistical analysis. CC, AI, MAC and MS drafted the manuscript. All authors critically reviewed the manuscript. All authors approved the final manuscript.

For peer review only

References

1. White GC, 2nd, Rosendaal F, Aledort LM, et al. Definitions in hemophilia. Recommendation of the scientific subcommittee on factor VIII and factor IX of the scientific and standardization committee of the International Society on Thrombosis and Haemostasis. *Thromb Haemost* 2001;85(3):560.
2. Avina-Zubieta JA, Galindo-Rodriguez G, Lavalle C. Rheumatic manifestations of hematologic disorders. *Curr Opin Rheumatol* 1998;10(1):86-90.
3. McCoy HE, 3rd, Kitchens CS. Small bowel hematoma in a hemophiliac as a cause of pseudoappendicitis: diagnosis by CT imaging. *Am J Hematol* 1991;38(2):138-9.
4. Fernandez-Palazzi F, Hernandez SR, De Bosch NB, et al. Hematomas within the iliopsoas muscles in hemophilic patients: the Latin American experience. *Clin Orthop Relat Res* 1996(328):19-24.
5. Small S, Rose PE, McMillan N, et al. Haemophilia and the kidney: assessment after 11-year follow-up. *Br Med J (Clin Res Ed)* 1982;285(6355):1609-11.
6. Ljung RC. Intracranial haemorrhage in haemophilia A and B. *Br J Haematol* 2008;140(4):378-84. doi: 10.1111/j.1365-2141.2007.06949.x
7. Darby SC, Kan SW, Spooner RJ, et al. Mortality rates, life expectancy, and causes of death in people with hemophilia A or B in the United Kingdom who were not infected with HIV. *Blood* 2007;110(3):815-25. doi: 10.1182/blood-2006-10-050435
8. Rodriguez-Merchan EC. Musculoskeletal complications of hemophilia. *HSS J* 2010;6(1):37-42. doi: 10.1007/s11420-009-9140-9
9. Lobet S, Hermans C, Lambert C. Optimal management of hemophilic arthropathy and hematomas. *J Blood Med* 2014;5:207-18. doi: 10.2147/JBM.S50644
10. Mazepa MA, Monahan PE, Baker JR, et al. Men with severe hemophilia in the United States: birth cohort analysis of a large national database. *Blood* 2016;127(24):3073-81. doi: 10.1182/blood-2015-10-675140
11. Tagliaferri A, Di Perna C, Santoro C, et al. Cancers in patients with hemophilia: a retrospective study from the Italian Association of Hemophilia Centers. *J Thromb Haemost* 2012;10(1):90-5. doi: 10.1111/j.1538-7836.2011.04566.x
12. Kulkarni R, Soucie JM, Evatt BL, et al. Prevalence and risk factors for heart disease among males with hemophilia. *Am J Hematol* 2005;79(1):36-42. doi: 10.1002/ajh.20339
13. Sharathkumar AA, Soucie JM, Trawinski B, et al. Prevalence and risk factors of cardiovascular disease (CVD) events among patients with haemophilia: experience of a single haemophilia treatment centre in the United States (US). *Haemophilia* 2011;17(4):597-604. doi: 10.1111/j.1365-2516.2010.02463.x
14. Ferreira AA, Leite IC, Bustamante-Teixeira MT, et al. Health-related quality of life in hemophilia: results of the Hemophilia-Specific Quality of Life Index (Haem-a-Qol) at a Brazilian blood center. *Rev Bras Hematol Hemoter* 2013;35(5):314-8. doi: 10.5581/1516-8484.20130108
15. Health USDo, Human Services FDACfDE, Research, et al. Guidance for industry: patient-reported outcome measures: use in medical product development to support labeling claims: draft guidance. *Health Qual Life Outcomes* 2006;4:79. doi: 10.1186/1477-7525-4-79

16. Deshpande PR, Rajan S, Sudeepthi BL, et al. Patient-reported outcomes: A new era in clinical research. *Perspect Clin Res* 2011;2(4):137-44. doi: 10.4103/2229-3485.86879
17. Lowe MM, Blaser DA, Cone L, et al. Increasing Patient Involvement in Drug Development. *Value Health* 2016;19(6):869-78. doi: 10.1016/j.jval.2016.04.009
18. Hoos A, Anderson J, Boutin M, et al. Partnering With Patients in the Development and Lifecycle of Medicines: A Call for Action. *Ther Innov Regul Sci* 2015;49(6):929-39. doi: 10.1177/2168479015580384
19. Skinner MW. WFH: closing the global gap--achieving optimal care. *Haemophilia* 2012;18 Suppl 4:1-12. doi: 10.1111/j.1365-2516.2012.02822.x
20. Noone D, O'Mahony B, van Dijk JP, et al. A survey of the outcome of prophylaxis, on-demand treatment or combined treatment in 18-35-year old men with severe haemophilia in six countries. *Haemophilia* 2013;19(1):44-50. doi: 10.1111/j.1365-2516.2012.02934.x
21. Benjamin K, Vernon MK, Patrick DL, et al. Patient-Reported Outcome and Observer-Reported Outcome Assessment in Rare Disease Clinical Trials: An ISPOR COA Emerging Good Practices Task Force Report. *Value Health* 2017;20(7):838-55. doi: 10.1016/j.jval.2017.05.015
22. Skinner MW, Driessens M, Curtis R, et al. The Patient Reported Outcomes, Burdens and Experiences (PROBE) Study Phase 1 Methodology and Feasibility. *Haemophilia* 2016;22 (Suppl. 4):118.
23. EuroQol G. EuroQol--a new facility for the measurement of health-related quality of life. *Health Policy* 1990;16(3):199-208.
24. Dyer JS, Rarin RK. Measurable multiattribute value functions. *Oper Res* 1979;27: 810 - 22.
25. Kirkwood CW. Strategic Decision Making: Multiobjective Decision Analysis with Spreadsheets Belmont, CA: Wadsworth Publ. Co

1996.
26. Chai-Adisaksopha C, Iorio A, Curtis R, et al. Test-Retest Reliability Analysis of The Patient Reported Outcomes Burdens and Experiences (PROBE) Study. *Haemophilia* 2017;23 (Suppl.2):29-140. [abstract P037]. doi: 10.1302/0301-620X.92B8.23922
27. Larsen R, Warne RT. Estimating confidence intervals for eigenvalues in exploratory factor analysis. *Behav Res Methods* 2010;42(3):871-6. doi: 10.3758/BRM.42.3.871
28. Cronbach LJ, Warrington WG. Time-limit tests: estimating their reliability and degree of speeding. *Psychometrika* 1951;16(2):167-88.
29. Evans JD. Straightforward statistics for the behavioral sciences. Pacific Grove, CA: Brooks/Cole Publishing. 1996.
30. Tabachnick BG, Fidell LS. Using multivariate statistics. Boston, MA: Allyn & Bacon 2007.
31. Herdman M, Gudex C, Lloyd A, et al. Development and preliminary testing of the new five-level version of EQ-5D (EQ-5D-5L). *Qual Life Res* 2011;20(10):1727-36. doi: 10.1007/s11136-011-9903-x
32. McLaughlin JM, Munn JE, Anderson TL, et al. Predictors of quality of life among adolescents and young adults with a bleeding disorder. *Health Qual Life Outcomes* 2017;15(1):67. doi: 10.1186/s12955-017-0643-7

For peer review only

33. Brown TM, Lee WC, Joshi AV, et al. Health-related quality of life and productivity impact in haemophilia patients with inhibitors. *Haemophilia* 2009;15(4):911-7. doi: 10.1111/j.1365-2516.2009.02032.x
34. Recht M, Neufeld EJ, Sharma VR, et al. Impact of acute bleeding on daily activities of patients with congenital hemophilia with inhibitors and their caregivers and families: observations from the Dosing Observational Study in Hemophilia (DOSE). *Value Health* 2014;17(6):744-8. doi: 10.1016/j.jval.2014.07.003
35. Scalone L, Mantovani LG, Mannucci PM, et al. Quality of life is associated to the orthopaedic status in haemophilic patients with inhibitors. *Haemophilia* 2006;12(2):154-62. doi: 10.1111/j.1365-2516.2006.01204.x
36. Feng Y, Devlin N, Herdman M. Assessing the health of the general population in England: how do the three- and five-level versions of EQ-5D compare? *Health Qual Life Outcomes* 2015;13:171. doi: 10.1186/s12955-015-0356-8

For peer review only

Figure legends

Figure 1 Flow diagram of participant selection

Figure 2. Scree plot of exploratory principal-component factors analysis

For peer review only

Table 1. Participants' characteristics

Characteristics	Participants (n=916)
Age, median (Q1, Q3)	37 (27, 48)
Diagnosis, n (%)	
• Hemophilia A	532 (58.1)
• Hemophilia B	82 (8.9)
• Non-hemophilia	302 (33.0)
Severity of hemophilia*, n (%)	
• Normal	3 (0.6)
• Mild	54 (10.6)
• Moderate	88 (17.3)
• Severe	352 (69.3)
• Do not know	11 (2.2)
Ever been diagnosed with inhibitor*, n (%)	
• Yes	70 (14.1)
• No	384 (77.2)
• Do not know	43 (8.7)
Currently have an clinically significant inhibitor, n (%)	24 (2.6)
Sex, n (%)	
• Male	685 (74.8)
• Female	231 (25.2)
Age when started school, median (Q1, Q3)	6 (5, 6)
Year of school or education, median (Q1, Q3)	15 (12, 18)
Married or long-term relationship, n (%)	581 (69.0)
Having Children, n (%)	462 (55.3)
Region, n (%)	
Africa	8 (0.9)
Western Pacific	216 (23.6)
South America	343 (37.4)
North America	138 (15.1)
Europe	211 (23.0)

*hemophilia population

Abbreviations: Q1; the first quartile, Q3; the third quartile

For peer review only

Table 2. Item distribution and missing data

Item	Floor (%)	Ceiling (%)	Missing (%)
Patient reported outcome			
Q.8 Problem related to health*	59.1	32.3	8.6
Q.9 Mobility aids or assistive devices	0.1	0	11.5
Q.10 Pain medications	3.0	14.6	12.3
Q.11.1 Acute pain (activities)	0.7	33.1	12.8
Q.11.2 Acute pain (interference)	0.3	33.2	12.8
Q.12.1 Chronic pain (activities)	1.4	32.6	13.5
Q.12.2 Chronic pain (interference)	0.1	33.6	13.5
Q.13 Daily activities	0.1	42.4	14.3
Q.14 Work/school life	0.1	27.8	21.8
Q.15 Joint surgery or procedure	1.3	52.4	17.0
Q.16 Comorbid diseases	0	56.1	0
Hemophilia related health			
Q.17 Severity	N/A	N/A	17.3
Q.18 Inhibitor status	N/A	N/A	19.1
Q.19 Bleeding in the past 12 months	16.6	8.5	18.2
Q.20 Bleeding in the past 2 weeks	N/A	N/A	18.9
Q.21 Hemophilia treatment center	N/A	N/A	19.4
Q.25 Target joints	N/A	N/A	22.6
Q. 26 spontaneous bleeding	N/A	N/A	49.4
Q.27 Limitation of range of motion*	66.6	11.4	22.0
Q.28 Life- or limb-threatening bleeding*	15.2	62.1	22.8
EQ-5D-5L and EQ-VAS			
Mobility	1.1	32.4	21.6
Self-care	0.7	55.0	22.3
Usual activities	0.7	37.9	22.4
Pain/discomfort	1.1	23.9	22.9
Anxiety/depression	1.6	37.3	22.8
VAS	0	3.1	22.8

*dichotomous outcome

N/A: not applicable

For peer review only

Table 3. Principal-component factors analysis, non-orthogonal rotated structure matrix loadings

Items	Factor1	Factor2	Uniqueness
Q.8 Problem related to health	**0.5648**	0.1011	0.6707
Q.9 Mobility aids or assistive devices	**0.4653**	-0.1721	0.7539
Q.10 Pain medications	**0.6571**	-0.0856	0.5609
Q.11.1 Acute pain (activities)	**0.7273**	-0.2825	0.3913
Q.11.2 Acute pain (interference)	**0.7275**	-0.3425	0.3535
Q.12.1 Chronic pain (activities)	**0.7853**	0.1408	0.3635
Q.12.2 Chronic pain (interference)	**0.8061**	0.1257	0.3344
Q.13 Daily activities	**0.7868**	0.0102	0.3808
Q.14 Work/school life	**0.5562**	-0.2130	0.6453
Q.15 Joint surgery or procedure	0.3142	**0.6981**	0.4139
Q.16 Comorbid diseases	0.4140	**0.5146**	0.5638

Table 4. Correlations between PROBE and EQ-5D-5L items (convergent validity)

EQ-5D-5L	PROBE	Correlation	95% confidence interval
Mobility	Q.9 Mobility aids	0.42	0.35 to 0.47
Pain and discomfort	Q.10 Pain medications	0.55	0.50-0.60
	Q.11.1 Acute pain (activities)	0.42	0.36 to 0.48
	Q.11.2 Acute pain (interference)	0.39	0.32 to 0.45
	Q.12.1 Chronic pain (activities)	0.56	0.51 to 0.61
	Q.12.2 Chronic pain (interference)	0.57	0.52 to 0.62
Self care	Q.13 Activities of daily living	0.65	0.61 to 0.69
Usual activities	Q.13 Activities of daily living	0.71	0.67 to 0.74
Anxiety	N/A	N/A	N/A
Utility index score	Total score	0.67	0.62 to 0.71

For peer review only

Table 5. Known group validity analyses, univariate analysis

Subgroup	Total PROBE score, mean (SD)	p-value
Q.2 Diagnosis<br>• Non-hemophilia<br>• Hemophilia	 0.87 (0.11)<br>0.71 (0.16)	 <0.001
Q.17 Severity of hemophilia<br>• Mild-moderate<br>• Severe	 0.71 (0.16)<br>0.70 (0.16)	 0.45
Q.18 Current inhibitor<br>• No<br>• Yes	 0.71 (0.19)<br>0.67 (0.12)	 0.35
Q.19 Number of bleeds in past year<br>• 0 bleed<br>• 1 bleed<br>• 2-3 bleeds<br>• 4-7 bleeds<br>• 8-10 bleeds<br>• 11-15 bleeds<br>• 16-30 bleeds<br>• >30 bleeds	 0.80 (0.14)<br>0.85 (0.11)<br>0.75 (0.15)<br>0.74 (0.14)<br>0.70 (0.13)<br>0.68 (0.12)<br>0.65 (0.15)<br>0.61 (0.15)	 <0.001
Q.20 Bleed in the past two weeks<br>• No<br>• Yes	 0.76 (0.15)<br>0.67 (0.15)	 <0.001
Q.25 Target joint<br>• No<br>• Yes	 0.78 (0.16)<br>0.68 (0.15)	 <0.001
Q.26 Spontaneous joint bleeding<br>• No<br>• Yes	 0.73 (0.15)<br>0.66 (0.14)	 0.0004
Q.27 having reduced range of motion<br>• No<br>• Yes	 0.86 (0.13)<br>0.68 (0.14)	 <0.001
Q.28 Life threatening bleed<br>• No<br>• Yes	 0.72 (0.15)<br>0.62 (0.16)	 <0.001

For peer review only

Table 6. Coefficients derived from multivariable linear regression analysis

	Coefficient*	95% confidence interval	p-value
Q.2 Diagnosis			
• Non-hemophilia	Control	N/A	N/A
• hemophilia	-0.22	-0.25 to -0.18	<0.001
Q.17 Severity of hemophilia			
• Mild-Moderate	Control	N/A	N/A
• Severe	-0.003	-0.03 to 0.03	0.83
Q.18 Current inhibitor			
• No	Control	N/A	N/A
• Yes	-0.04	-0.14 to 0.05	0.34
Q.19 Number of bleeds in past year			
• 0 bleed	Control	N/A	N/A
• 1 bleed	0.04	-0.03 to 0.10	0.29
• 2-3 bleeds	-0.06	-0.11 to 0.001	0.06
• 4-7 bleeds	-0.07	-0.12 to -0.01	0.02
• 8-10 bleeds	-0.10	-0.16 to -0.03	0.002
• 11-15 bleeds	-0.14	-0.20 to 0.08	<0.001
• 16-30 bleeds	-0.15	-0.21 to -0.09	<0.001
• >30 bleeds	-0.19	-0.24 to -0.13	<0.001
Q.20 Bleed in the past two weeks			
• No	Control	N/A	N/A
• Yes	-0.09	-0.12 to -0.07	<0.001
Q.25 Target joint			
• No	Control	N/A	N/A
• Yes	-0.09	-0.13 to -0.06	<0.001
Q.26 Spontaneous joint bleeding			
• No	Control	N/A	N/A
• Yes	-0.09	-0.12 to -0.05	<0.001
Q.27 having reduced range of motion			
• No	Control	N/A	N/A
• Yes	-0.14	-0.19 to -0.11	<0.001
Q.28 Life threatening bleed			
• No	Control	N/A	N/A
• Yes	-0.10	-0.13 to -0.06	<0.001

*Adjusted from age and sex

Abbreviation: N/A; not applicable

For peer review only

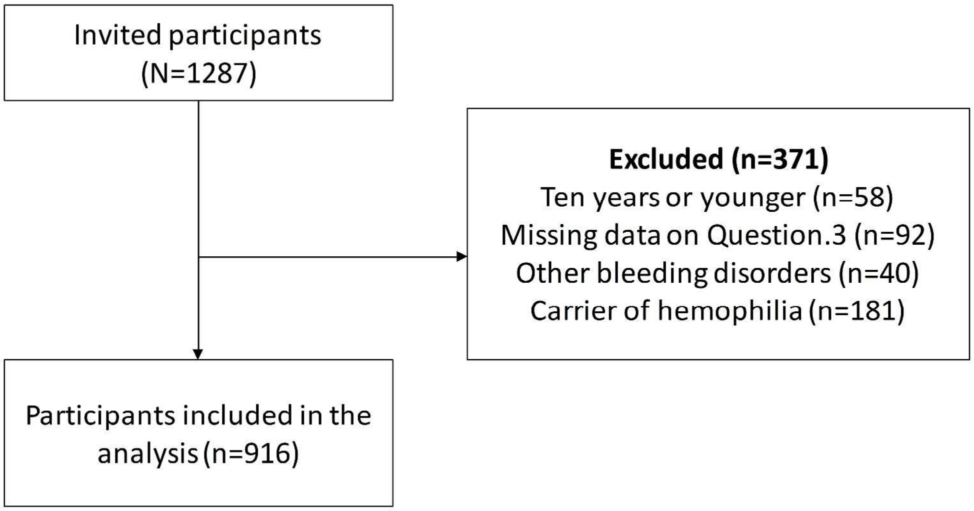

Figure 1 Flow diagram

er review only

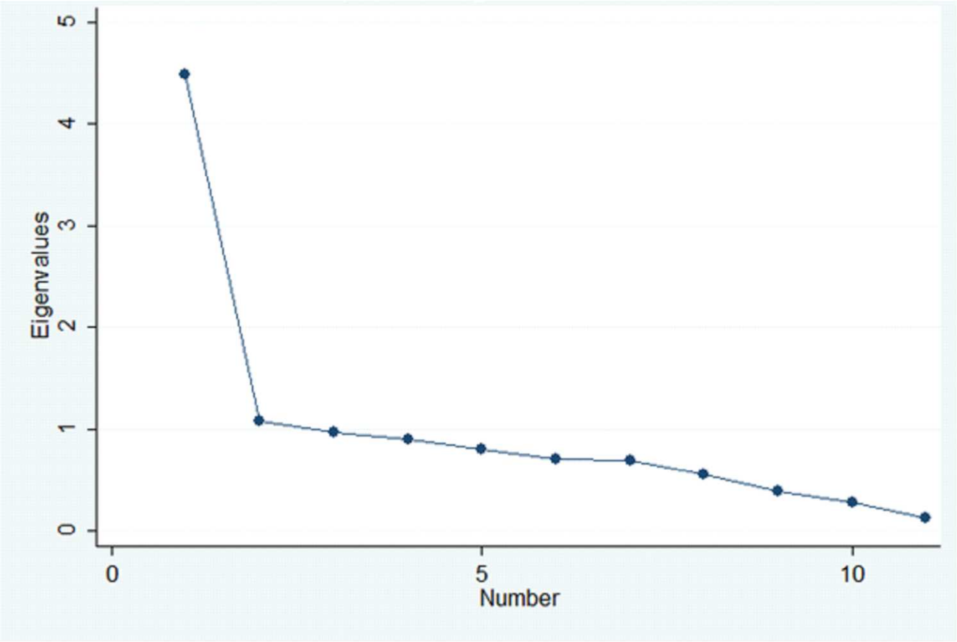

Figure 2 Scree plot

BMJ Open

Psychometric properties of the Patient Reported Outcomes Burdens and Experiences (PROBE) Questionnaire

Journal:	*BMJ Open*
Manuscript ID	bmjopen-2018-021900.R1
Article Type:	Research
Date Submitted by the Author:	23-Apr-2018
Complete List of Authors:	Chai-Adisaksopha, Chatree; McMaster University, Medicine; McMaster University, Health Research Methods, Evidence, and Impact<br>Skinner, Mark; Institute for Policy Advancement Ltd<br>Curtis, Randall; Factor VIII Computing<br>Frick, Neil; National Hemophilia Foundation<br>Nichol, Michael; University of Southern California Sol Price School of Public Policy<br>Noone, Declan; Irish Haemophilia Society<br>O'Mahony, Brian ; Irish Haemophilia Society; Trinity College Dublin<br>Page, David; Canadian Hemophilia Society<br>Stonebraker, Jeffrey ; North Carolina State University Poole College of Management<br>Thabane, Lehana; McMaster University, Health Research Methods, Evidence, and Impact; St Joseph's Healthcare, Biostatistics Unit<br>Crowther, Mark; McMaster University, Canada<br>Iorio, Alfonso; McMaster University, Clinical Epidemiology and Biostatistics
**Primary Subject Heading**:	Patient-centred medicine
Secondary Subject Heading:	Haematology (incl blood transfusion)
Keywords:	validity, hemophilia, patient reported outcome, quality of life, questionnaire

SCHOLARONE™
Manuscripts

Psychometric properties of the Patient Reported Outcomes Burdens and Experiences (PROBE) Questionnaire

Authors

Chatree Chai-Adisaksopha[1,2], Mark Skinner[3], Randall Curtis[4], Neil Frick[5], Michael B. Nichol[6], Declan Noone[7], Brian O'Mahony[7,8], David Page[9], Jeffrey Stonebraker[10], Lehana Thabane[2,11], Mark Crowther[1,2] and Alfonso Iorio[1,2]

Authors' affiliations

[1]Department of Medicine, McMaster University, Hamilton, ON, Canada

[2]Department of Health Research Methods, Evidence, and Impact, McMaster University, Hamilton, Canada

[3]Institute for Policy Advancement Ltd, Washington, DC, US

[4]Factor VIII Computing, Berkeley, US

[5]National Hemophilia Foundation, New York, US

[6]Sol Price School of Public Policy, University of Southern California, Los Angeles, US

[7]Irish Haemophilia Society, Dublin, Ireland

[8]Trinity College Dublin, Dublin, Ireland

[9]Canadian Hemophilia Society, Montreal, Canada

[10]Poole College of Management, North Carolina State University, Raleigh, US

[11]Biostatistics Unit, St Joseph's Healthcare, Hamilton, Canada

For peer review only

Sources of financial support: PROBE is an independent investigator led research project with grant / research support from: Baxalta, now part of Shire; Bayer; Bioverativ; CSL Behring; Novo Nordisk, Roche and Sobi with administrative support provided by the US National Hemophilia Foundation.

Acknowledgements: We thank the hemophilia patient organizations that have participated in the PROBE Project: Fundación de la Hemofilia (Argentina) Cordoba Chapter; Hemophilia Foundation Australia (Australia); Federaçao Brasileira de Hemofilia (Brazil); Canadian Hemophilia Society (Canada); Association Française des Hémophiles (France); Deutsche Hämophiliegesellschaft (Germany); Magyar Hemofilia Egyesulet (Hungary); Irish Haemophilia Society (Ireland); Federazione delle Associazioni Emofilici (Italy); National Hemophilia Network of Japan (Japan); Federación de Hemofilia de la República Mexicana (Mexico); Nederlandse Vereniging van Hemofilie- Patiënten (The Netherlands); Haemophilia Foundation of New Zealand (New Zealand); Haemophilia Foundation of Nigeria (Nigeria); Polish Hemophilia Society (Poland), Federación Española de Hemofilia (Spain); The Haemophilia Society (United Kingdom); National Hemophilia Foundation (United States); Asociación Venezolana para la Hemofilia (Venezuela) and Vietnamese Hemophilia Association (Vietnam). We also thank two anonymous reviewers for significant contribution to the quality and clarity of the final version of this manuscript, including some key methodological references.

Running title: Psychometric properties of PROBE

Keywords: validity, hemophilia, patient reported outcome, quality of life, questionnaire

Word count: Abstract 245, Text 3715

Table and Figure: 6 tables, 1 figure

For peer review only

Correspondence:

Mark W. Skinner

1155 23rd Street NW #3A

Washington DC 20037 USA

Email: mskinnerdc@gmail.com

Tel:. +1-202-253-8342

Fax: +1-202-296-1352

For peer review only

Abstract

Objective: To assess the psychometric properties of The Patient Reported Outcomes, Burdens and Experiences (PROBE) questionnaire.

Methods: This study was a cross-sectional, multi-national study. Participants were enrolled if they were 10 years or older and patients with hemophilia A or B or people without bleeding disorder. Participants were invited through non-governmental patient organizations in 21 countries between 04/08/2015 and 12/28/2015.The following psychometric properties: missing data, floor and ceiling effects, exploratory factor analysis, and internal consistency reliability were examined. A PROBE Score was derived and assessed for its convergent and known groups validity.

Results: The study analyzed the data on 916 participants with median age of 37.0 (interquartile range 27.0 to 48.0) years, 74.8% male. In the domain assessing patient reported outcomes, more than 15% of participants presented a ceiling effect for all items but two, and a floor effect for one item. Factor analysis identified two factors explaining the majority of the variance. Cronbach's alpha coefficient indicated good internal consistency reliability (0.84). PROBE items showed moderate to strong correlations with corresponding EQ-5D-5L domains. The PROBE Score has a strong correlation (r=0.67) with EQ-5D-5L utility index score. The PROBE Score has a known groups validity among various groups.

Conclusions: The results of this study suggest that PROBE is a valid questionnaire for evaluating PROs in people with hemophilia, as well as control population. The known-group property of PROBE will allow its use in future clinical trials, longitudinal studies, health technology assessment studies, routine clinical care or registries. Additional studies are needed to test responsiveness and sensitivity to change.

Trial registration: NCT02439710

For peer review only

Strengths and limitation

- The PROBE questionnaire was conducted to assess patient reported outcomes in people with hemophilia (PWH). This tool assesses domains pertaining to general health status, hemophilia related health status and health-related quality of life.
- The psychometric analyses demonstrate the validity and internal consistency of the Patient Reported Outcomes Burdens and Experiences (PROBE) Questionnaire.
- This study was conducted in a large sample of PWH and participants without bleeding disorders from multiple countries.
- The responsiveness of the measurement was not investigated in this current study.

For peer review only

Background

Hemophilia is an inherited X-linked recessive bleeding disorder characterized by the reduction or absence of blood coagulation factor (F) VIII (hemophilia A) or FIX (hemophilia B). Severity of hemophilia is categorized by the baseline factor level (mild; factor level >0.05 to <0.40 IU/ml, moderate; factor level 0.01-0.05 IU/ml and severe; factor level<0.01 IU/ml) [1]. Coagulation deficiency renders patients prone to abnormal bleeding. Symptoms of hemophilia vary depending on the severity of hemophilia, mechanism and severity of injury and affected organs. People with hemophilia (PWH) commonly present with hemarthrosis, gastrointestinal or genitourinary tract bleeding, intramuscular bleeding or intracranial bleeding [2-6].

Life expectancy of PWH substantially improved with factor replacement therapy [7]. However, PWH who live longer encounter more chronic complications from both hemophilia-related conditions and degenerative diseases that occur in normal population. Chronic degenerative joint diseases are found in 90% of PWH by the second or third decade of life [8]. PWH with recurrent joint bleeding suffer from chronic pain, limitation of range of motion and disability [9]. Human immunodeficiency virus (HIV) and hepatitis C virus (HCV) infections are prevalent among PWH prior to the implementation of intensive viral screening in plasma-derived factor concentrates and the use of recombinant factor concentrates [10]. One of the major consequences of chronic HCV infection is cirrhosis, resulting in end-stage liver disease which is the most common cause of death in PWH [10]. Moreover, 43% of cancers diagnosed in PWH were related to HCV infection [11]. Aged PWH are also affected by cardiovascular diseases. A retrospective study using an administrative database of 3,422 males with hemophilia reported a prevalence of ischemic heart disease of 15% in PWH older than 60 years [12]. Risk factors of

For peer review only

cardiovascular disease in PWH are equivalent to patients without hemophilia [13]. These long-term complications of hemophilia directly impact on health-related quality of life (HRQoL) in PWH [14].

Patient reported outcomes (PROs) are defined as any reports of status of patients' health conditions that come directly from the patients without interpretation by clinicians or anyone else [15]. PROs provide data obtained from patients including symptoms, frequency of symptoms, severity of symptoms, impact of disease on daily life, disability and perfection of patients toward diseases and treatments [16]. Thus, PROs have been increasingly valued by researchers, stakeholders, policy makers and health technology assessment agencies [17-20]. Recently, the International Society for Pharmacoeconomic and Outcomes Research (ISPOR) Clinical Outcome Assessment Emerging Good Practices Task Force published the Patient-reported outcome and observer-reported outcome assessment in rare disease clinical trials [21]. This report demonstrated the challenges of assessing patient-reported outcomes in rare diseases, for instance, heterogeneity of disease severity and patient experience or understanding treatment benefit from the patients' perspective. Hemophilia, which is a rare bleeding disorder, exhibits various disease severity. Moreover, patients' perspective on their symptoms may be dissimilarly influenced by age, co-morbid disease, inhibitor status, current treatment or progression of symptoms. Therefore, a hemophilia-specific PRO measure is essential for assessing outcomes in this patient population.

The Patient Reported Outcomes, Burdens and Experiences (PROBE) Project is a patient-lead research initiative. The main objectives of the PROBE Project are to develop a standardised PRO questionnaire and to develop a dedicated research network to generate and continuously update PROBE reference data. The feasibility study of the PROBE questionnaire was

For peer review only

conducted in collaborations with non-governmental hemophilia patient organizations (NGOs) in 21 countries. Previously reported results demonstrated that the burden of the PROBE questionnaire implementation was minimal and the time required to complete the questionnaire was less than 15 minutes for over 75% of participants [22]. The objective of the current study is to assess the psychometric properties of the PROBE questionnaire.

For peer review only

Methods

Patient and public involvement

The PROBE Project was initiated and led by investigators who are patients with hemophilia. Subsequently, the investigators identified and invited a group of national hemophilia patient organizations to participate in the PROBE Project to form a research network. The patient-important outcomes and metrics incorporated into the PROBE questionnaire were identified, developed, and refined by the PROBE investigators and patient representatives from the participating national patient organizations (see acknowledgments). The patient organization were then asked to enroll participants. Data from the PROBE study are analyzed, summarized and disseminated to each patient organization. Full development details of the PROBE questionnaire and patient-led research network are reported elsewhere[23].

Participant enrollment and study procedure

This study was designed as a cross-sectional assessment. Participants were enrolled through NGOs from 1/27/2016 to 2/23/2017. Participants were recruited if they were more than 10 years old and they were either PWH (hemophilia A or hemophilia B) or controls (participants without bleeding disorders). Participants were instructed to complete the questionnaire only once and answering for themselves, and parents or caregivers were instructed not to answer for their child. Although collected as part of the study, participants who identified themselves as carriers of hemophilia were excluded from the analysis. Patients with other bleeding disorders or an unknown bleeding disorder were also excluded. Participants who did not respond to Q.3 (hemophilia diagnosis: hemophilia A, hemophilia B, no bleeding disorder) were excluded from the analysis. The participating NGOs distributed the PROBE questionnaires through mail, e-mail, in-person meetings or a combination of methods. The

For peer review only

PROBE questionnaire was available in 18 languages with localized language versions in both paper- and web-based format. A central statistical check for duplicates was run, and 3 potential duplicates were excluded.

Ethical approval

Patients' identifier or personal information were not collected as part of the study. Data were collected as anonymous individuals, and study data were transferred and stored at McMaster University. Ethical approval was obtained from the Hamilton Integrated Research Ethics Boards. Additional local review ethical board approval was obtained when requested by the local regulation.

PROBE questionnaire

The detail of questionnaire development and feasibility study was described elsewhere [22]. The PROBE questionnaire is organized in 4 sections, comprising 29 questions. Sections are numbered following the order of presentation in the questionnaire. PROBE PRO domains are covered in Section II. The questions in Section I and III do not cover PRO domains. Only PWH are expected to complete Section III, whereas every participant completes Sections I, II and IV. Section I contains 7 questions pertaining to demographic data (country, gender, diagnosis of hemophilia or absence of a bleeding disorder, year of birth, body weight, age first started and finished school, marital status and children). Section II contains 9 questions pertaining to PROs, including general health issues, use of mobility aids or assistive devices, pain (including acute, chronic, and pain medications), daily activities, current work or student status, surgeries or procedures, and co-morbid diseases. Section III contains 12 questions pertaining to clinical aspects of hemophilia (severity of hemophilia, inhibitor status, bleeding history, hemophilia care, treatment regimen, target joints, joint bleeding, range of motion and

For peer review only

life- or limb-threatening bleeds). Section IV contains the EuroQol five dimension 5-level instrument (EQ-5D-5L) [24], consisting of questions regarding mobility, self-care, usual activities, pain or discomfort and anxiety or depression, and the EuroQol visual analog scale (EQ-VAS) of global health [24] were incorporated in the PROBE questionnaire with permission.

Item scaling and PROBE score calculation

PROs were evaluated only in Section II. The calculation of the PROBE score was based on multiattribute value functions [25 26]. The assessed scores (X_i) were converted to returns-to-scale score (V_iX_i), given that $0 \leq V_i(X_i) \leq 1$. Q.8 which had a dichotomous response (0 = no, 1 = yes) produce dichotomous score of 0 and 1. Two questions (Q.10 and Q.15) asked for frequency of the use of pain medication(s) and number of surgeries or invasive procedures. The 6- and 7-level Likert scales from these two questions were converted to a returns-to-scale score, ranging from 0 to 1. The number of days absent from work or school (Q.14) was converted to returns-to-scale score by dividing by 366. Questions regarding mobility aids, acute pain, chronic pain and co-morbid diseases (Q.9, Q.11, Q.12, Q.13 and Q.16) had multiple choices. The scales for these items were calculated based on the cumulative number of choices checked. We apply weight for subitems in each question (if needed). The final score was calculated by summing all of the 11 items scores from the 9 questions using additive value function and then scaled so the PROBE Score ranged from 0 to 1 (higher value indicates better health status).

Data analyses

Descriptive statistics

Demographic data of study participants were summarized using mean with corresponding standard deviation (SD) or median and quartile range as appropriate. Categorical data were

For peer review only

summarized using numbers and percentages. Participants who did not respond in Q.3 (disease status; hemophilia A, hemophilia B, hemophilia carrier, other bleeding disorders or no bleeding disorder) were excluded from the analysis. An item distribution analysis to evaluate the proportion of missing data was performed. Floor and ceiling effects were evaluated by the proportion of respondents with scores at floor (minimum score) and ceiling (maximum score), respectively. We pre-defined that we would have considered a floor or ceiling effect relevant using the empirical threshold of 15% and a cumulative ceiling or flooring of 50% as proposed by Terwee et al[27].

Psychometric analyses

Face and content validity were assessed and reported previously [22]. Test-retest reliability analyses of the PROBE questionnaire were reported elsewhere [28]. In the current study, the following psychometric analyses were carried out.

Principal axis factor analysis

An exploratory factor analysis of 9 questions, pertaining to the PROs (Section II). Principal axis factor analysis with oblique rotation method was performed. The percentage of variance on the items that were explained by the factors was evaluated. Higher percentage indicated strong influence of the factors. The regression coefficients (factor loadings) of the item responses on the retaining factors after factor rotation was calculated.

Internal consistency reliability

An analysis to confirm the precision of the scale based on the intercorrelations of the items evaluating the same construct was conducted. We hypothesized that the questions asking about pain and the use of medications (Q.10-Q.13) were correlated. Cronbach's alpha was

For peer review only - http://bmjopen.bmj.com/site/about/guidelines.xhtml

used to determine the correlation between items. Cronbach's alpha coefficient greater than 0.7 was considered to indicate acceptable reliability [29].

Convergent validity

The convergent validity of the items in the same construct with the existing, standardised questionnaire were assessed. Specifically, we hypothesized that the items asking about the use of mobility aids and assistive devices correlated with the mobility domain of EQ-5D-5L; the items asking about the use of pain medication, acute and chronic pain (Q.10, Q.11 and Q.12) correlated with pain and discomfort domain of EQ-5D-5L; the items asking about activities of daily living (Q.13) correlated with the self-care and usual activity domains of EQ-5D-5L[30]. Each item of EQ-5D-5L was scored, ranging from level 1 (coded as 1) to level 5 (coded as 5). The health states were converted into a single index value utilizing the United Kingdom value set. The correlation between the score from each PROBE item and corresponding EQ-5D-5L domain was calculated. Additionally, the correlation between EQ-5D-5L utility index score and the PROBE Score was assessed. Correlation coefficient (r) was interpreted as: r 0.20-0.39; weak correlation; r 0.40-0.59, moderate correlation; r 0.60-0.79, strong correlation; and r 0.80-1.00, very strong correlation [31].

Known groups validity

The ability of the PROBE questionnaire to determine the differences between known subgroups was assessed. Participants were classified into groups, according to information collected in Section III, as diagnosis (hemophilia or non-hemophilia), severity of hemophilia (mild, moderate or severe), current inhibitor status (yes or no), number of bleeds in the past year (categorical variable), bleed in the past two weeks (yes, no), presence of target joint (yes, no), limitation of range of motion of the joints (yes, no) and life- or limb-threatening bleeding

For peer review only

in the past year (yes, no). The PROBE Scores were compared between subgroups using t-test or one-way ANOVA for the univariate analysis, as appropriate. A priori hypotheses included PWH (as compared to participants without bleeding disorders), patients with severe hemophilia (as compared to mild and moderate hemophilia), patients with current inhibitor (as compared to those without an inhibitor), patients with greater numbers of bleeding, patients who had recent bleeding within the past 2 weeks (as compared to those without), patients with presence of target joint(s) (as compared to those without), patients who had reduced range of motion of any joints (as compared to those without) and patients who had life- or limb-threatening bleeding in the past year (as compared to those without) had worse PROBE scores. The multivariable analysis of the known group validity was conducted using a linear regression. The regression model included age and gender of participants in the analysis. Regression coefficients with corresponding 95% CI were reported. P-value less than 0.05 was considered statistically significant.

For peer review only

Results

Participants' demographic data

Since inception, NGOs from 21 countries have participated in the PROBE Project. For this study, we performed the analysis using participants' data from the first 17 countries. Figure 1 demonstrates the flow of participant selection for this phase of research. There were 1287 participants who responded to the questionnaire. After excluding hemophilia carriers, other bleeding disorders and missing value (Question 3), and 3 possible duplicates, the analysis included 916 participants. Demographic data is shown in Table 1. Median age of PWHs was lower than that of controls, 33 (quartile 1, quartile 3 of 24, 46) vs 43 (quartile 1, quartile 3 of 34, 54) years. The proportion of male participants in hemophilia group was greater than those in control group (93.7% vs 6.4%). Among hemophilia patients, most had severe hemophilia. Seventeen participants (2.6%) of PWH had an inhibitor during the study period.

Descriptive analysis

Table 2 demonstrates item distribution and missing data. Ceiling effect greater than 15% was observed in all but one item (the use of pain medications) in Section II. Similarly, ceiling effect greater than 15% was observed in all domains of EQ-5D-5L. Floor effect greater than 15% was found in four items (problems related to health, bleeding in the past 12 months, limitation of range of motion and life- or limb-threatening bleeding). We observed a higher frequency of ceiling effect among participants without a bleeding disorder as compared to PWH (data not shown). Missing data was 0% to 21.8% in Section II, 18.2% to 49.4% in Section III and 21.6% to 22.9% in Section IV. The median PROBE Score across all participants was 0.78 (mean=0.76, SD=0.16, minimum=0.26 and maximum=0.99).

Principal axis factor analysis

For peer review only

The principal component factor analysis of the 9 questions (11 items) pertaining to the PROs was carried out. These three factors were retained for the following analyses. Table 3 demonstrates factor loadings based on three factors. The items were grouped per factor with their maximum loading (bold).

Factor 1 appears to be the most influential, explaining 87.3% of the variance. There were two items contained in this factor (activities and interference related to chronic pain). Factor 2 contained two items (activities and interference related to acute pain). Factor 3 contained 2 items pertaining to daily activities and work/school life. All items in the each factor had acceptable factor loadings ($r \geq 0.3$) [32].

Internal consistency reliability

The Cronbach's alpha coefficient was acceptable at 0.84.

Convergent validity

Table 4 shows the correlation coefficients between PROBE items and EQ-5D-5L. The results showed that Q. 9 (the use of mobility aids and assistive devices) had a moderate correlation with mobility domain of EQ-5D-5L (r=0.42). The pain and discomfort domain of EQ-5D-5L had a moderate to strong correlation with most of the pain related items of the PROBE questionnaire (r=0.55 for pain medication, 0.42 for acute pain occurrence, 0.39 for acute pain interference, 0.56 for chronic pain occurrence and 0.57 for chronic pain interference). Item related to activities of daily living had a strong correlation with the self care and usual activities domain (r=0.65 and 0.71, respectively). The PROBE score had a strong correlation with the EQ-5D-5L utility index score (r=0.67).

Known groups validity

For peer review only - http://bmjopen.bmj.com/site/about/guidelines.xhtml

The regression coefficients of each *a priori* variable and the PROBE Score were demonstrated in Table 5. Participants without a bleeding disorder had a significantly higher PROBE Score when compared with PWH (mean score (SD), 0.87 (0.11) vs 0.71 (0.16), P<0.001). PWH with mild to moderate hemophilia had a slightly higher PROBE Score (mean 0.71, SD 0.16) than severe PWH (mean 0.70, SD 0.16), PWH who had a greater number of bleeding episodes had a significantly lower PROBE Score when compared to those who had less frequent bleeding (P<0.001). Patients who reported bleeding in the past two weeks had a significantly lower PROBE score (mean 0.67, SD 0.15) than those without (mean 0.76, SD 0.15). Patients who reported the presence of any target joints had a significantly lower PROBE score (mean 0.68, SD 0.15) when compared to those who did not (mean 0.78, SD 0.16). Patients who reported three or more spontaneous joint bleeds in the past 6 months had significantly lower PROBE score (mean 0.66, SD 0.14) than those who did not report (mean 0.73, SD 0.14). Patients with reduced range of motion of any joints had a significantly lower PROBE score (mean 0.68, SD 0.14) as compared to those without (mean 0.73, SD 0.15). Patients who previously had life- or limb-threatening bleeding in the past year had a significantly lower PROBE Score (mean 0.62, SD 0.16) when compared to those who did not (mean 0.72, SD 0.15). Table 6 demonstrates multivariable analysis. The findings from multivariable analysis did not change much after adjusting for age and sex.

For peer review only

Discussion

The psychometric properties of the PROBE questionnaire have been assessed, and found that the PROBE questionnaire has a strong internal consistency, robust convergent validity and excellent differentiation properties between known groups. We believe these characteristics, jointly with the availability of country specific reference ranges and low impact on NGO resources and time required by the patients make the PROBE questionnaire a tool with great potential for efficient PROs collection in clinical and comparative effectiveness research, and for advocacy purposes.

As demonstrated by factor analysis, the core of PROBE revolves around two factors, explaining the majority of the variance in responses. The most influential factor was pain, followed by use of mobility aids or assistive device (complemented by work or school absent days), and comorbidity. No surprise these three elements explain 50% of the variance among different participants: the novelty of PROBE is summarizing the assessment of these 3 domains in a lightweight set of questions for which excellent internal consistency was demonstrated.

The convergent validity analysis showed moderate to strong correlation between PROBE and EQ-5D-5L items, with lower correlations for items concerning pain (*r* ranged from 0.39 to 0.57). Whereas the overall convergence with EQ-5D-5L was confirmed, and was intentionally sought to ensure maximizing external validity and efficiency for cross-disease comparisons. The pain related questions in the PROBE questionnaire are related to different aspects (when the pain occurred..., if the pain interfered with any of following…) than EQ-5D-5L [33]. From this perspective, PROBE might be seen as a new hybrid PRO tool, sharing some properties of a generic and some of a disease specific tool. The total PROBE score has a strong correlation

with the utility index score of the EQ-5D-5L, both in patients (*r*=0.57), and controls (*r*=0.53), but explores a more specific set of subdomains.

The most important result of this analysis is the demonstration of the discriminative property of the PROBE questionnaire and score. In known group validity analysis, PWH had a significantly lower PROBE Score when compared to the control population (participants without hemophilia). Patients with more frequent bleeds, target joints, reduced range of motion and previous life- or limb-threatening bleeds were demonstrated with a lower PROBE score (indicating worse health status).

The investigators did not observe a significant difference of the total PROBE scores among severity of disease, as well as, current inhibitor status. This outcome may be confounded by bleeding phenotype and joint status. It has been shown that the presence of inhibitor has negative impact on health-related quality of life in PWH [34]. The regression analysis in this present study revealed that numbers of bleeding, presence of target joint(s) and limitation of range of motion of any joints, not inhibitor status, were associated with worse health status. There have been studies that reported the negative health-related quality of life in hemophilia patients with inhibitor who had poor orthopedic joint score, who had acute bleeding and who had more frequent bleeding [35-37]. It is important to note that there are relatively a small number of patients with mild-moderate diseases (8.8% and 14.3%, respectively) and those with current inhibitors (4.1%) in this study. The association between inhibitor status and health status of PWH warrant further studies with adequate power.

The PROBE Project has several strengths. First, both PWH and participants without bleeding disorders were recruited, asked PRO questions meaningful to both, and derived a PROBE score applicable to both. Therefore, we were able to compare the health status across health-

For peer review only

specific conditions (hemophilia vs non-hemophilia in this study). There is a potential role for the use of the PROBE questionnaire to compare health status between PWH with any other diseases that share common features, e.g. von Willebrand disease, rheumatoid arthritis or osteoarthritis. Second, both school-aged and adult participants were included. The work or school life was assessed in the same manner. As a result, the PROBE questionnaire is valid to implement in participants in all age groups (starting at the not-yet defined age when one is able to comprehend the questionnaire). Third, the questions in the PROBE questionnaire included a standardized observation period in each question stem, generally the past 12 months. This is helpful for participants to respond to each item closest to their actual health condition in a specific time frame.

This PROBE Project also has some limitations, the first being that responsiveness of the PROBE Score has not been validated currently. This study was conducted with a cross-sectional study design. This means participants responded to the questionnaire at a single time. Assessing responsiveness requires a more complicated and demanding study design, which will be addressed in the future. Second, the observation period in the items was up to 12 months. Whereas this was chosen to maximize capturing the impact of rare events, it might introduce recall bias in some participants. Third, a ceiling effect was observed for all except one item concerning PRO, as well as, all EQ-5D-5L items. The recent study regarding floor and ceiling effects of the EQ-5D-5L in 996 English general population showed that 47.6% of respondents reported the best possible heath state (ceiling effect) [38]. In addition, the ceiling effects ranged from 58.4% to 90.8% in the subdomains [38]. The floor effects in the study were relatively lower than the previous reports [38], probably because sicker participants (PWH) were included

For peer review only

Conclusions

The psychometric properties of the PROBE questionnaire have been assessed, showing that the PROBE questionnaire has a strong internal consistency, robust convergent validity and excellent differentiation properties between known groups. When compared to EQ-5D-5L, PROBE has a moderate to strong correlation across all domains. The immediate use of the PROBE score based on these results would be in cross-sectional comparisons among different settings, e.g. those defined by different levels of access to care. The PROBE questionnaire has great potential for efficient PROs collection in clinical and comparative effectiveness research, and for advocacy purposes. Future applications of PROBE within clinical trials or in longitudinal observational studies will require preliminary demonstration of PROBE test-retest and responsiveness properties, to ensure it is sensitive to meaningful treatment or disease changes over time.

For peer review only

List of abbreviations

PROBE: Psychometric properties of the Patient Reported Outcomes Burdens and Experiences; EQ-5D-5L: EuroQol five dimension 5-level instrument; F:factor; EQ-VAS: EuroQol visual analog scale; PWH: people with hemophilia; HIV: Human immunodeficiency virus; HCV: hepatitis C virus; HRQoL: health-related quality of life; PRO: Patient reported outcome; ISPOR: International Society for Pharmacoeconomic and Outcomes Research; NGO: non-governmental organization; SD: standard deviation; ANOVA; analysis of variance

Declarations

Ethics approval and consent to participate

Ethical approval was obtained from the Hamilton Integrated Research Ethics Boards. Additional local review ethical board approval was obtained when requested by the local regulation.

Consent for publication

Not Applicable.

Availability of data and material

Not Applicable.

Competing interests

For peer review only

CC, LT, MAC have no potential conflict of interest. Investigators received grants from Baxalta, now part of Shire; Bayer; Bioverativ; CSL Behring, Novo Nordisk; Roche; and Sobi and non-financial support from the US National Hemophilia Foundation.

Funding

PROBE is an independent investigator led research project with grant / research support from: Baxalta, now part of Shire; Bayer; Bioverativ; CSL Behring; Novo Nordisk, Roche and Sobi with administrative support provided by the US National Hemophilia Foundation.

Acknowledgements: The PROBE investigators wish to thank the hemophilia patient organizations that have participated in the project and two anonymous reviewers for significant contribution to the quality and clarity of the final version of this manuscript, including some key methodological references.

Authors' contributions

MS, AI, RC, NF, MN, DN, BOM, DP and JS conceptualized the study. CC and LT performed data collection and statistical analysis. CC, AI, MAC and MS drafted the manuscript. All authors critically reviewed the manuscript. All authors approved the final manuscript.

References

For peer review only

1. White GC, 2nd, Rosendaal F, Aledort LM, et al. Definitions in hemophilia. Recommendation of the scientific subcommittee on factor VIII and factor IX of the scientific and standardization committee of the International Society on Thrombosis and Haemostasis. *Thromb Haemost* 2001;85(3):560.
2. Avina-Zubieta JA, Galindo-Rodriguez G, Lavalle C. Rheumatic manifestations of hematologic disorders. *Curr Opin Rheumatol* 1998;10(1):86-90.
3. McCoy HE, 3rd, Kitchens CS. Small bowel hematoma in a hemophiliac as a cause of pseudoappendicitis: diagnosis by CT imaging. *Am J Hematol* 1991;38(2):138-9.
4. Fernandez-Palazzi F, Hernandez SR, De Bosch NB, et al. Hematomas within the iliopsoas muscles in hemophilic patients: the Latin American experience. *Clin Orthop Relat Res* 1996(328):19-24.
5. Small S, Rose PE, McMillan N, et al. Haemophilia and the kidney: assessment after 11-year follow-up. *Br Med J (Clin Res Ed)* 1982;285(6355):1609-11.
6. Ljung RC. Intracranial haemorrhage in haemophilia A and B. *Br J Haematol* 2008;140(4):378-84. doi: 10.1111/j.1365-2141.2007.06949.x
7. Darby SC, Kan SW, Spooner RJ, et al. Mortality rates, life expectancy, and causes of death in people with hemophilia A or B in the United Kingdom who were not infected with HIV. *Blood* 2007;110(3):815-25. doi: 10.1182/blood-2006-10-050435
8. Rodriguez-Merchan EC. Musculoskeletal complications of hemophilia. *HSS J* 2010;6(1):37-42. doi: 10.1007/s11420-009-9140-9
9. Lobet S, Hermans C, Lambert C. Optimal management of hemophilic arthropathy and hematomas. *J Blood Med* 2014;5:207-18. doi: 10.2147/JBM.S50644
10. Mazepa MA, Monahan PE, Baker JR, et al. Men with severe hemophilia in the United States: birth cohort analysis of a large national database. *Blood* 2016;127(24):3073-81. doi: 10.1182/blood-2015-10-675140
11. Tagliaferri A, Di Perna C, Santoro C, et al. Cancers in patients with hemophilia: a retrospective study from the Italian Association of Hemophilia Centers. *J Thromb Haemost* 2012;10(1):90-5. doi: 10.1111/j.1538-7836.2011.04566.x
12. Kulkarni R, Soucie JM, Evatt BL, et al. Prevalence and risk factors for heart disease among males with hemophilia. *Am J Hematol* 2005;79(1):36-42. doi: 10.1002/ajh.20339
13. Sharathkumar AA, Soucie JM, Trawinski B, et al. Prevalence and risk factors of cardiovascular disease (CVD) events among patients with haemophilia: experience of a single haemophilia treatment centre in the United States (US). *Haemophilia* 2011;17(4):597-604. doi: 10.1111/j.1365-2516.2010.02463.x
14. Ferreira AA, Leite IC, Bustamante-Teixeira MT, et al. Health-related quality of life in hemophilia: results of the Hemophilia-Specific Quality of Life Index (Haem-a-Qol) at a Brazilian blood center. *Rev Bras Hematol Hemoter* 2013;35(5):314-8. doi: 10.5581/1516-8484.20130108
15. Health USDo, Human Services FDACfDE, Research, et al. Guidance for industry: patient-reported outcome measures: use in medical product development to support labeling claims: draft guidance. *Health Qual Life Outcomes* 2006;4:79. doi: 10.1186/1477-7525-4-79
16. Deshpande PR, Rajan S, Sudeepthi BL, et al. Patient-reported outcomes: A new era in clinical research. *Perspect Clin Res* 2011;2(4):137-44. doi: 10.4103/2229-3485.86879
17. Lowe MM, Blaser DA, Cone L, et al. Increasing Patient Involvement in Drug Development. *Value Health* 2016;19(6):869-78. doi: 10.1016/j.jval.2016.04.009
18. Hoos A, Anderson J, Boutin M, et al. Partnering With Patients in the Development and Lifecycle of Medicines: A Call for Action. *Ther Innov Regul Sci* 2015;49(6):929-39. doi: 10.1177/2168479015580384
19. Skinner MW. WFH: closing the global gap--achieving optimal care. *Haemophilia* 2012;18 Suppl 4:1-12. doi: 10.1111/j.1365-2516.2012.02822.x

For peer review only

20. Noone D, O'Mahony B, van Dijk JP, et al. A survey of the outcome of prophylaxis, on-demand treatment or combined treatment in 18-35-year old men with severe haemophilia in six countries. *Haemophilia* 2013;19(1):44-50. doi: 10.1111/j.1365-2516.2012.02934.x
21. Benjamin K, Vernon MK, Patrick DL, et al. Patient-Reported Outcome and Observer-Reported Outcome Assessment in Rare Disease Clinical Trials: An ISPOR COA Emerging Good Practices Task Force Report. *Value Health* 2017;20(7):838-55. doi: 10.1016/j.jval.2017.05.015
22. Skinner MW, Driessens M, Curtis R, et al. The Patient Reported Outcomes, Burdens and Experiences (PROBE) Study Phase 1 Methodology and Feasibility. *Haemophilia* 2016;22 (Suppl. 4):118.
23. Skinner MW, Chai-Adisaksopha C, Curtis R, et al. The Patient Reported Outcomes, Burdens and Experiences (PROBE) Project: development and evaluation of a questionnaire assessing patient reported outcomes in people with haemophilia. *Pilot Feasibility Stud* 2018;4:58. doi: 10.1186/s40814-018-0253-0
24. EuroQol G. EuroQol--a new facility for the measurement of health-related quality of life. *Health Policy* 1990;16(3):199-208.
25. Dyer JS, Rarin RK. Measurable multiattribute value functions. *Oper Res* 1979;27: 810 - 22.
26. Kirkwood CW. Strategic Decision Making: Multiobjective Decision Analysis with Spreadsheets Belmont, CA: Wadsworth Publ. Co 1996.
27. Terwee CB, Bot SD, de Boer MR, et al. Quality criteria were proposed for measurement properties of health status questionnaires. *J Clin Epidemiol* 2007;60(1):34-42. doi: 10.1016/j.jclinepi.2006.03.012
28. Chai-Adisaksopha C, Iorio A, Curtis R, et al. Test-Retest Reliability Analysis of The Patient Reported Outcomes Burdens and Experiences (PROBE) Study. *Haemophilia* 2017;23 (Suppl.2):29-140. [abstract P037]. doi: 10.1302/0301-620X.92B8.23922
29. Cronbach LJ, Warrington WG. Time-limit tests: estimating their reliability and degree of speeding. *Psychometrika* 1951;16(2):167-88.
30. Hoots WK, Ebbesen LS, Konkle BA, et al. Secondary prophylaxis with recombinant activated factor VII improves health-related quality of life of haemophilia patients with inhibitors. *Haemophilia* 2008;14(3):466-75. doi: 10.1111/j.1365-2516.2008.01654.x
31. Evans JD. Straightforward statistics for the behavioral sciences. Pacific Grove, CA: Brooks/Cole Publishing. 1996.
32. Tabachnick BG, Fidell LS. Using multivariate statistics. Boston, MA: Allyn & Bacon 2007.
33. Herdman M, Gudex C, Lloyd A, et al. Development and preliminary testing of the new five-level version of EQ-5D (EQ-5D-5L). *Qual Life Res* 2011;20(10):1727-36. doi: 10.1007/s11136-011-9903-x
34. McLaughlin JM, Munn JE, Anderson TL, et al. Predictors of quality of life among adolescents and young adults with a bleeding disorder. *Health Qual Life Outcomes* 2017;15(1):67. doi: 10.1186/s12955-017-0643-7
35. Brown TM, Lee WC, Joshi AV, et al. Health-related quality of life and productivity impact in haemophilia patients with inhibitors. *Haemophilia* 2009;15(4):911-7. doi: 10.1111/j.1365-2516.2009.02032.x
36. Recht M, Neufeld EJ, Sharma VR, et al. Impact of acute bleeding on daily activities of patients with congenital hemophilia with inhibitors and their caregivers and families: observations from the Dosing Observational Study in Hemophilia (DOSE). *Value Health* 2014;17(6):744-8. doi: 10.1016/j.jval.2014.07.003
37. Scalone L, Mantovani LG, Mannucci PM, et al. Quality of life is associated to the orthopaedic status in haemophilic patients with inhibitors. *Haemophilia* 2006;12(2):154-62. doi: 10.1111/j.1365-2516.2006.01204.x

For peer review only

38. Feng Y, Devlin N, Herdman M. Assessing the health of the general population in England: how do the three- and five-level versions of EQ-5D compare? *Health Qual Life Outcomes* 2015;13:171. doi: 10.1186/s12955-015-0356-8

For peer review only

Figure legends

Figure 1 Flow diagram of participant selection

Table 1. Participants' characteristics

For peer review only

Characteristics	Participants (n=916)#
Age, median (Q1, Q3)	37 (27, 48)
Diagnosis, n (%)	
• Hemophilia A	532 (58.1)
• Hemophilia B	82 (8.9)
• Non-hemophilia	302 (33.0)
Severity of hemophilia*, n (%)	
• Normal	3 (0.6)
• Mild	54 (10.6)
• Moderate	88 (17.3)
• Severe	352 (69.3)
• Do not know	11 (2.2)
Ever been diagnosed with inhibitor*, n (%)	
• Yes	70 (14.1)
• No	384 (77.2)
• Do not know	43 (8.7)
Currently have an clinically significant inhibitor, n (%)	24 (2.6)
Sex, n (%)	
• Male	685 (74.8)
• Female	231 (25.2)
Age when started school, median (Q1, Q3)	6 (5, 6)
Year of school or education, median (Q1, Q3)	15 (12, 18)
Married or long-term relationship, n (%)	581 (69.0)
Having Children, n (%)	462 (55.3)
Region, n (%)	
Africa	8 (0.9)
Western Pacific	216 (23.6)
South America	343 (37.4)
North America	138 (15.1)
Europe	211 (23.0)

after exclusion of 3 possible duplicates

*hemophilia population

Abbreviations: Q1; the first quartile, Q3; the third quartile

For peer review only

Table 2. Item distribution and missing data

Item	Floor (%)	Ceiling (%)	Missing (%)
Patient reported outcome			
Q.8 Problem related to health*	59.1	32.3	8.6
Q.9 Mobility aids or assistive devices	0.1	0	11.5
Q.10 Pain medications	3.0	14.6	12.3
Q.11.1 Acute pain (activities)	0.7	33.1	12.8
Q.11.2 Acute pain (interference)	0.3	33.2	12.8
Q.12.1 Chronic pain (activities)	1.4	32.6	13.5
Q.12.2 Chronic pain (interference)	0.1	33.6	13.5
Q.13 Daily activities	0.1	42.4	14.3
Q.14 Work/school life	0.1	27.8	21.8
Q.15 Joint surgery or procedure	1.3	52.4	17.0
Q.16 Comorbid diseases	0	56.1	0
Hemophilia related health			
Q.17 Severity	N/A	N/A	17.3
Q.18 Inhibitor status	N/A	N/A	19.1
Q.19 Bleeding in the past 12 months	16.6	8.5	18.2
Q.20 Bleeding in the past 2 weeks	N/A	N/A	18.9
Q.21 Hemophilia treatment center	N/A	N/A	19.4
Q.25 Target joints	N/A	N/A	22.6
Q. 26 spontaneous bleeding	N/A	N/A	49.4
Q.27 Limitation of range of motion*	66.6	11.4	22.0
Q.28 Life- or limb-threatening bleeding*	15.2	62.1	22.8
EQ-5D-5L and EQ-VAS			
Mobility	1.1	32.4	21.6
Self-care	0.7	55.0	22.3
Usual activities	0.7	37.9	22.4
Pain/discomfort	1.1	23.9	22.9
Anxiety/depression	1.6	37.3	22.8
VAS	0	3.1	22.8

*dichotomous outcome

N/A: not applicable

For peer review only

1 Table 3. Principal axis factor analysis, non-orthogonal rotated structure matrix loadings

Items	Factor1	Factor2	Factor3	Uniqueness
Q.8 Problem related to health	0.1053	0.1416	0.0277	0.7022
Q.9 Mobility aids or assistive devices	-0.1540	0.0442	0.3470	0.7427
Q.10 Pain medications	0.2065	0.0684	0.1394	0.6174
Q.11.1 Acute pain (activities)	-0.0033	**0.7963**	0.0158	0.3111
Q.11.2 Acute pain (interference)	0.0763	**0.7701**	0.0005	0.2900
Q.12.1 Chronic pain (activities)	**0.8214**	0.0386	0.0329	0.2128
Q.12.2 Chronic pain (interference)	**0.8315**	0.0152	0.0092	0.1969
Q.13 Daily activities	0.2573	0.0229	**0.5321**	0.3854
Q.14 Work/school life	0.0679	0.0477	**0.5931**	0.6613
Q.15 Joint surgery or procedure	0.0489	0.0222	-0.0031	0.8356
Q.16 Comorbid diseases	-0.0022	-0.0832	0.0642	0.7874

2

3

4 Table 4. Correlations between PROBE and EQ-5D-5L items (convergent validity)

EQ-5D-5L	PROBE	Correlation	95% confidence interval
Mobility	Q.9 Mobility aids	0.42	0.35-0.47
Pain and discomfort	Q.10 Pain medications	0.55	0.50-0.60
	Q.11.1 Acute pain (activities)	0.42	0.36-0.48
	Q.11.2 Acute pain (interference)	0.39	0.32-0.45
	Q.12.1 Chronic pain (activities)	0.56	0.51-0.61
	Q.12.2 Chronic pain (interference)	0.57	0.52-0.62
Self-care	Q.13 Activities of daily living	0.65	0.61-0.69
Usual activities	Q.13 Activities of daily living	0.71	0.67-0.74
Anxiety	N/A	N/A	N/A
Utility index score	Total score	0.67	0.62-0.71

5 Abbreviation: N/A; not applicable

6

For peer review only

Table 5. Known group validity analyses, univariate analysis

Subgroup	Total PROBE score, mean (SD)	p-value
Q.2 Diagnosis		
• Non-hemophilia	0.87 (0.11)	<0.001
• Hemophilia	0.71 (0.16)	
Q.17 Severity of hemophilia		
• Mild-moderate	0.71 (0.16)	0.45
• Severe	0.70 (0.16)	
Q.18 Current inhibitor		
• No	0.71 (0.19)	0.35
• Yes	0.67 (0.12)	
Q.19 Number of bleeds in past year		
• 0 bleed	0.80 (0.14)	<0.001
• 1 bleed	0.85 (0.11)	
• 2-3 bleeds	0.75 (0.15)	
• 4-7 bleeds	0.74 (0.14)	
• 8-10 bleeds	0.70 (0.13)	
• 11-15 bleeds	0.68 (0.12)	
• 16-30 bleeds	0.65 (0.15)	
• >30 bleeds	0.61 (0.15)	
Q.20 Bleed in the past two weeks		
• No	0.76 (0.15)	<0.001
• Yes	0.67 (0.15)	
Q.25 Target joint		
• No	0.78 (0.16)	<0.001
• Yes	0.68 (0.15)	
Q.26 Spontaneous joint bleeding		
• No	0.73 (0.15)	0.0004
• Yes	0.66 (0.14)	
Q.27 having reduced range of motion		
• No	0.86 (0.13)	<0.001
• Yes	0.68 (0.14)	
Q.28 Life threatening bleed		
• No	0.72 (0.15)	<0.001
• Yes	0.62 (0.16)	

For peer review only

Table 6. Coefficients derived from multivariable linear regression analysis

	Coefficient*	95% confidence interval	p-value
Q.2 Diagnosis			
• Non-hemophilia	Control	N/A	N/A
• hemophilia	-0.22	-0.25 to -0.18	<0.001
Q.17 Severity of hemophilia			
• Mild-Moderate	Control	N/A	N/A
• Severe	-0.003	-0.03 to 0.03	0.83
Q.18 Current inhibitor			
• No	Control	N/A	N/A
• Yes	-0.04	-0.14 to 0.05	0.34
Q.19 Number of bleeds in past year			
• 0 bleed	Control	N/A	N/A
• 1 bleed	0.04	-0.03 to 0.10	0.29
• 2-3 bleeds	-0.06	-0.11 to 0.001	0.06
• 4-7 bleeds	-0.07	-0.12 to -0.01	0.02
• 8-10 bleeds	-0.10	-0.16 to -0.03	0.002
• 11-15 bleeds	-0.14	-0.20 to 0.08	<0.001
• 16-30 bleeds	-0.15	-0.21 to -0.09	<0.001
• >30 bleeds	-0.19	-0.24 to -0.13	<0.001
Q.20 Bleed in the past two weeks			
• No	Control	N/A	N/A
• Yes	-0.09	-0.12 to -0.07	<0.001
Q.25 Target joint			
• No	Control	N/A	N/A
• Yes	-0.09	-0.13 to -0.06	<0.001
Q.26 Spontaneous joint bleeding			
• No	Control	N/A	N/A
• Yes	-0.09	-0.12 to -0.05	<0.001
Q.27 having reduced range of motion			
• No	Control	N/A	N/A
• Yes	-0.14	-0.19 to -0.11	<0.001
Q.28 Life threatening bleed			
• No	Control	N/A	N/A
• Yes	-0.10	-0.13 to -0.06	<0.001

*Adjusted from age and sex

Abbreviation: N/A; not applicable

For peer review only

For peer review only - http://bmjopen.bmj.com/site/about/guidelines.xhtml

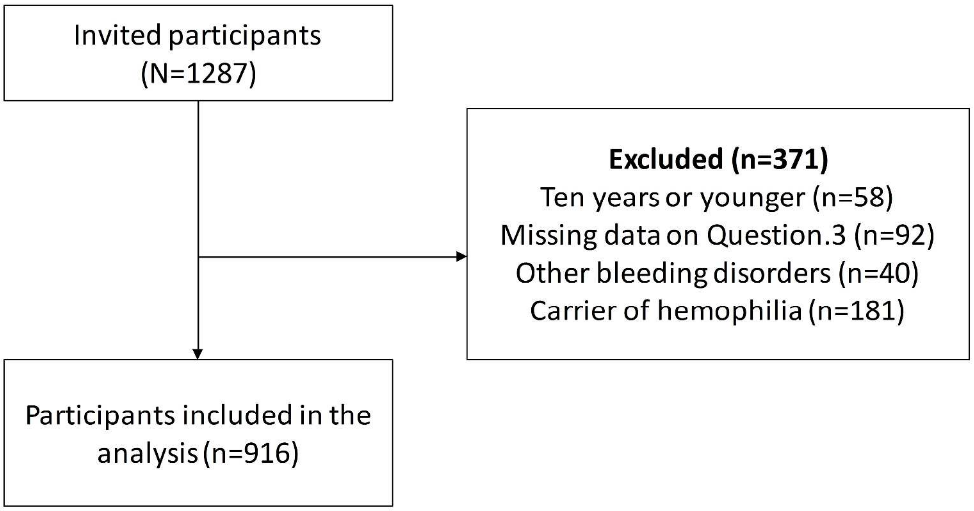

Figure 1 Flow diagram
